# The Safety and Effectiveness of Bevacizumab in Metastatic Colorectal Cancer With Unresectable Metastases: A Real-Life Study From the South of Morocco

**DOI:** 10.7759/cureus.56733

**Published:** 2024-03-22

**Authors:** Ghizlane Rais, Farah Boutaagount, Rania Mokfi, Meryem Maskrout, Soundous Bennour, Chaymae Senoussi, Fadoua Rais, Laila Lahlou

**Affiliations:** 1 Medical Oncology, Centre Hospitalier Universitaire (CHU) Souss Massa, Agadir, MAR; 2 Medical Oncology, Faculty of Medicine and Pharmacy of Agadir, Ibn Zohr University, Agadir, MAR; 3 Medical Oncology, Faculty of Medicine and Pharmacy of Agadir, University Ibn Zohr, Centre Hospitalier Universitaire (CHU) Souss Massa, Agadir, MAR; 4 Medical Oncology, Faculty of Medicine and Pharmacy of Agadir, University Ibn Zohr, CHU Souss MassaCentre Hospitalier Universitaire (CHU) Souss Massa, Agadir, MAR; 5 Radiation Therapy, University Hospital Center of Montreal, Montreal, CAN; 6 Epidemiology and Clinical Research Laboratory, Faculty of Medicine and Pharmacy of Agadir, Ibn Zohr University, Agadir, MAR

**Keywords:** effectiveness, unresectable metastases, safety, bevacizumab, metastatic colorectal cancer

## Abstract

Background

Colorectal cancer constitutes a significant public health challenge, despite remarkable strides made in the last two decades, particularly in the medical management of metastatic stages. Notable progress has been achieved through targeted therapies such as anti-epidermal growth factor receptors or anti-angiogenic antibodies, as well as advancements in surgical approaches for hepatic metastases. This study seeks to assess the efficacy and safety of bevacizumab plus chemotherapy in individuals with metastatic colorectal cancer.

Methodology

This is an observational, cross-sectional, retrospective study of all patients who were followed up for metastatic colorectal cancer with unresectable metastases and were treated with bevacizumab in combination with standard chemotherapy from January 2010 until December 2019 in the medical oncology department of the Centre Hospitalier Universitaire (CHU) Souss-Massa of Agadir.

Results

Of the total 162 cases, 117 (72%) had metastatic disease, and 45 (28%) progressed to metastatic disease after initial treatment. The median age of the patients was 55 years (range = 23-79 years) with a sex ratio of 1.1 (M/F). The tumor was located in the left colon in 135 (83.3%) patients. The results represented adenocarcinoma in 137 (84.6) cases and mucinous subtype in 23 (14.19) cases. The three most common sites of metastasis were the liver (99, 61.1), peritoneum (67, 41.3), and lung (33, 20.3). In the first line, all patients received bi-chemotherapy plus bevacizumab, i.e., fluorouracil, oxaliplatin, and leucovorin in 34 (20.9%) patients; capecitabine plus oxaliplatin in 88 (54.3%) patients; leucovorin, fluorouracil, and irinotecan in 17 (10.4%) patients; and capecitabine plus irinotecan in 23 (14.1%) patients. Response after first-line treatment was progression in 74 (45.7) cases, stability in 42 (25.9) cases, partial response in 35 (21.6) cases, and complete response in 11 (6.8) cases. Nine (6%) patients were able to benefit from surgical resection of metastatic lesions. Overall, 77 (47%) patients received second-line chemotherapy, i.e., 5-FU with irinotecan in 40 (51.8%) cases or with oxaliplatin in 30 (38.8%) cases. Two patients developed undesirable side effects under bevacizumab (hypertension). The median progression-free survival and median overall survival of the study cohort were 9 months and 14 months, respectively. Nevertheless, patients who underwent primary tumor resection (p = 0.048), those with right‑sided tumors (p = 0.022), those who received a higher number of treatment cycles (p = 0.020), and those who received maintenance treatment (p = 0.001) had a longer median overall survival.

Conclusions

Chemotherapy combination with bevacizumab is considered as the cornerstone of metastatic colorectal cancer treatment in our region. With the new healthcare and social security systems, easier access to expensive treatments and molecular pathology tests is currently available. It is important to highlight that real-world data can offer valuable insights into the daily clinical practice of medical oncology.

## Introduction

Colorectal cancer (CRC) is generally recognized as a common cancer and ranks second in terms of cancer-related deaths [1]. In Morocco, it is the third most common among cancer types based on data from regional registries, with its incidence showing an upward trend [2]. Over many years, there have been significant advancements and changes in the treatment landscape for metastatic colorectal cancer (mCRC). These improvements can be attributed to several factors, including advancements in liver surgery and interventional radiology that now allow for liver metastasectomy which was previously considered impossible. Moreover, valuable knowledge on how to combine and use cytotoxic agents has been gained from numerous multicenter studies [3]. Additionally, targeted treatments have played a role in these advancements by identifying molecular structures. Some notable examples include bevacizumab, a humanized monoclonal antibody targeting vascular endothelial growth factor, and panitumumab or cetuximab monoclonal antibodies targeting epidermal growth factor receptor (EGFR) [4]. These new therapies can now be used as an option for treating mCRC. In Morocco, the Ministry of Health and the Lalla Salma Foundation have collaborated to secure access to various expensive targeted drugs, including bevacizumab, for patients covered by public health insurance. However, this initiative has not been extended to cetuximab and panitumumab. Additionally, research on the *RAS*/*RAF* mutation has proven to be financially challenging for patients in this region, making access to these therapies more difficult. Hence, we conducted this study among patients being treated for mCRC, the first of its kind from southern Morocco.

The main aims of our study are to delineate patient characteristics, assess the utilization of bevacizumab and its effectiveness in extending progression-free survival (PFS) and overall survival (OS), identify predictive factors influencing outcomes, and finally examine safety in patients with mCRC undergoing treatment in routine medical practice.

This article was previously posted to the Research Square preprint server on February 1st, 2024. (https://www.researchsquare.com/article/rs-3910738/v1).

## Materials and methods

This was an observational, cross-sectional, retrospective study. We analyzed records of patients who were diagnosed with mCRC. This study was conducted at the Department of Medical Oncology in Souss Massa University Hospital Center of Agadir in Morocco from January 2010 to December 2019. This study was carried out with the approval of the local hospital committee, and data were collected by reviewing the hospital’s electronic database and patients’ medical records. Our study strictly adhered to the ethical guidelines for medical research involving human subjects, as outlined in the Declaration of Helsinki issued by the World Medical Association. The study included patients who met specific eligibility criteria, which included a histopathological diagnosis of CRC with metastatic disease and unresectable metastases. These eligible patients underwent treatment with bevacizumab in combination with standard chemotherapy. Patients who had undergone a minimum of one chemotherapy cycle were enrolled in this study. Conversely, the study excluded individuals who received supportive care, chemotherapy alone, or radiation therapy. For categorization purposes, tumors located in the rectosigmoid, descending colon, and splenic flexure were classified as left-sided colon cancers. Meanwhile, tumors located in the ascending colon were categorized as right-sided colon cancers. During the initial treatment phase, patients received first-line chemotherapy with bevacizumab, administered either at a dose of 5 mg/kg every two weeks or 7.5 mg/kg every three weeks. This was associated with chemotherapy based on either 5-FU or capecitabine in combination with oxaliplatin (fluorouracil, oxaliplatin, and leucovorin (FOLFOX), capecitabine plus oxaliplatin (XELOX)) or with irinotecan (leucovorin, fluorouracil, and irinotecan (FOLFIRI) capecitabine plus irinotecan (XELERI)). Afterward, maintenance treatment involved the continuation of bevacizumab in combination with chemotherapy.

Treatment efficacy was assessed through clinical examinations, carcinoembryonic antigen levels, and contrast-enhanced computed tomography (CECT) scans interpreted following the RECIST guidelines (version 1.1). These evaluations were conducted after a minimum of three to four cycles (approximately three months) of treatment.

Gathered data included patient demographics, disease specificities, treatment details, disease evolution, and mortality. Safety assessment focused on recognizing adverse events related to bevacizumab encompassing proteinuria, hypertension, bleeding, bowel perforation, impaired wound healing, and arterial thromboembolisms. Several factors were analyzed to determine the treatment outcomes, including age, gender, primary tumor location (right or left colon), surgical resection of metastatic lesions, and treatment response.

OS refers to the duration from the time of diagnosis of mCRC with CECT to the time of death. PFS delineates the time from the diagnosis to progression, the date of death, or the last day of follow-up. The scheduled follow-up date was January 30, 2022.

The Kaplan-Meier method was utilized to estimate PFS and OS, and comparisons were made using the log-rank test. A p-value <0.05 was considered statistically significant. To determine the associated factors for OS and PFS, we employed Cox proportional hazards models in both univariate and multivariate analyses. The factors with a significant p-value were introduced into the multivariate model. Then, we forced the model by introducing the variable (side) into the multivariate analysis of OS because it was described in another series.

## Results

Sociodemographic data

Between January 2010 and December 2019, a total of 162 patients were enrolled. The median age of our patients was 55 years (range = 23-79 years). Overall, 22.6% were categorized as young patients, aged 45 years or younger. The sex ratio was 1.1 (M/F), with 87 (54%) male and 75 (46%) female patients.

Regarding comorbid conditions, only 13 (8.1%) patients had additional health issues, which included diabetes (8, 4.9%), hypertension (3, 1.8%), and heart disease (2, 1.2%). Additionally, two patients had a familial history of cancer (Table 1).

**Table 1:** Patient demographics. The data are expressed using N values (%) and mean ± SD.

Patient characteristics (n = 162)	N (%)
Gender	
Female	75 (46)
Male	87 (54)
Age (mean ± SD)	55 ± 12.1 years (range = 23–79 years)
Age groups (years)	
≤45	37 (22.6)
46–69	110 (68.2)
≥70	15 (9.2)
Comorbid conditions	
Yes	13 (8.1)
No	149(91.9)
Type of comorbidities	
Hypertension	3 (1.8)
Heart disease	2 (1.2)
Diabetes	8 (4.9)

Clinical characteristics

Regarding tumor location, 80 (49.3%) patients had tumors in the left colon, 27 (16.6%) in the right colon, and 55 (33.9%) in the rectum. *RAS* mutational profiling was conducted for three patients in our study. Among the cases of CRC, 137 (84.6%) were classified as adenocarcinoma, and 23 (14.19%) as a mucinous subtype. Additionally, there were two cases with a kitten-ring cell component.

Of all our patients, 117 (72%) had de-novo metastatic disease, while 45 (28%) patients experienced disease progression leading to metastasis after their initial treatment.

The most prevalent metastasis sites were the liver, observed in 99 (61.1%) patients, followed by peritoneum in 67 (41.3%), lungs in 33 (20.3%), non-regional lymph nodes in 21 (12.9%), and, less commonly, ovaries in six (3.7%) and bone in three (1.8%) (Table 2).

**Table 2:** Clinical characteristics of the study participants. The data are expressed using N values (%).

Patient characteristics	N (%)	
Primary tumor site		
Left colon	80 (49.3)	
Right colon	27 (16.6)	
Rectum	55 (33.9)	
*RAS *status availability		
Yes	3 (1.8)	
No	159 (98.2)	
Morphology		
Well-differentiated adenocarcinoma	87 (53.7)	
Moderately differentiated adenocarcinoma	42 (25.9)	
Undifferentiated adenocarcinoma	8 (4.9)	
Signet ring	2 (1.2)	
Mucinous	23 (14.19)	
Patients		
de novo metastatic disease	117 (72)	
Progressed to metastatic disease after initial treatment	45 (28)	
Metastasis		
Liver	99 (61.1)	
Peritoneum	67 (41.3)	
Lung	33 (20.3)	
Non-regional lymph nodes	21 (12.9)	
Ovary	6 (3.7)	
Bone	3 (1.8)	
Number of organs involved		
1	103 (63.5)	
2	47 (29.0)	
≥3	11 (6.7)	

Treatment and safety

In the first-line setting, all patients received a chemotherapy combination regimen associated with bevacizumab. Overall, 122 (75%) patients received oxaliplatin-based chemotherapy with FOLFOX in 34 (20.9%) and XELOX in 88 (54.3%) patients, and 40 (25%) patients received irinotecan-based chemotherapy with FOLFIRI in 17 (10.4%) and XELERI in 23 (14.1%) (Table 3). The median number of received cycles was six. After first-line chemotherapy, the observed response was progression in 74 (45.7) cases, stability in 42 (25.9) cases, and either partial or complete response in 35 (21.6) and 11 (6.8) cases, respectively (Table 4). Nine (6%) patients had metastatic resection after first-line chemotherapy. Overall, 77 (47%) patients received second-line chemotherapy based on 5-FU with irinotecan in 40 (51.8%) cases or with oxaliplatin in 30 (38.8%) cases (Table 3). Regarding side effects, two patients developed bevacizumab-related adverse events (hypertension).

**Table 3:** Chemotherapy regimen given in first- and second-line settings. The data are expressed using N values (%). FOLFOX = fluorouracil, oxaliplatin, and leucovorin; XELOX = capecitabine plus oxaliplatin; FOLFIRI = leucovorin, fluorouracil, and irinotecan; XELERI = capecitabine plus  irinotecan; GEMOX = gemcitabine and oxaliplatin

Chemotherapy lines	Regimen	N (%)
First-line (N = 162)	FOLFOX	34 (20.9)
	XELOX	88 (54.3)
	FOLFIRI	17 (10.4)
	XELERI	23 (14.1)
Second-line (N = 77)	FOLFOX	4 (5.1)
	XELOX	26 (33.7)
	FOLFIRI	16 (20.7)
	XELERI	24 (31.1)
	GEMOX	7 (9.0)

**Table 4:** Response rate to first- and second-line chemotherapy. The data are expressed using N values (%).

Response	First line, N = 162 (%)	Second line, N = 77 (%)
Progression	74 (45.7)	45 (58.9)
Stability	42 (25.9)	16 (21.4)
Partial response	35 (21.6)	13 (14.3)
Complete response	11 (6.8)	4 (5.4 )

Survival analysis

**Figure 1:**
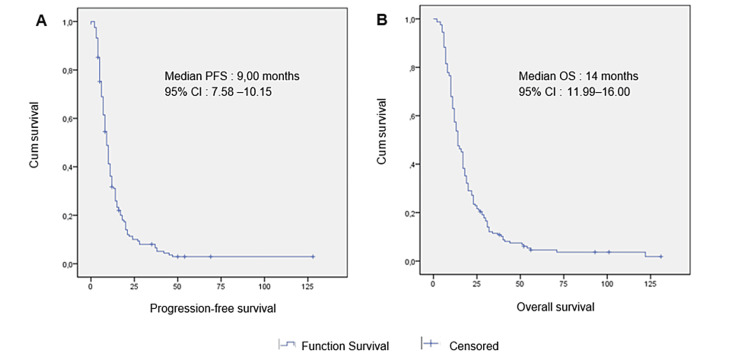
Progression-free survival (A) and overall survival (B) for the entire population.

The median PFS for patients receiving bevacizumab as first-line treatment was nine months (95% confidence interval (CI) = 7.58-10.15) (Figure 1A). Regarding PFS, factors that showed significance during univariate analysis included primary tumor resection and the number of chemotherapy cycles. However, no differences were found according to age, gender, side, or chemotherapy regimen. Multivariate analysis revealed that two factors remained significantly associated with a lower PFS, namely, patients without primary tumor resection and patients who received a lower number of treatment cycles (Table 5). The median OS for the study cohort was 14 months (95% CI = 11.99-16.00) (Figure 1B). No significant disparity was observed in the median OS based on gender, age (either less or more than 45 years old), chemotherapy regimen, and liver or peritoneum metastasis. Nevertheless, patients who underwent primary tumor resection (p = 0.048), those with right‑sided tumors (p = 0.022), those who received a higher number of treatment cycles (p = 0.020), and those who received maintenance treatment (p = 0.001) had a longer median OS (Table 6).

**Table 5:** Univariate and multivariate analyses of different prognostic factors for progression-free survival. The p-value is deemed significant at <0.05.

Variable		Univariate analysis		Multivariate analysis	
		Hazard ratio (CI)	P-value	Hazard ratio (CI)	P-value
Age	(<45 years vs. ≥45 years)	1.424 (0.966–2.098)	0.074	-	
Gender	(Female vs. Male)	0.978 (0.708–1.351)	0.891	-	-
Tumor location	(Left-sided vs. Right-sided)	0.828 (0.539–1.271)	0.389	-	-
Chemotherapy regimen	(Irinotecan vs. Oxali-based)	1.233 (0.848–1.794)	0.273	-	-
Number of cycles	-	0.757 (0.668–0.858)	0.002	0.758 (0.669–0.860)	0.001
Peritoneum metastasis	(Yes vs. No)	1.328 (0.958–1.840)	0.89	-	-
Liver metastasis	(Yes vs. No)	1.255 (0.899–1.752)	0.182	-	-
Resection of the primary tumor	(Yes vs. No)	0.313 (0.144–0.678)	0.003	0.197 (0.086–0.451)	0.0001

**Table 6:** Univariate and multivariate analyses of different prognostic factors for overall survival. The p-value is deemed significant at <0.05.

Variable		Univariate analysis		Multivariate analysis	
		Hazard ratio	P-value	Hazard ratio	P-value
Gender	(Female vs. Male)	0.946 (0.686–1.304)	0.735	-	-
Age	(<45 years vs. ≥45 years)	1.310 (0.896–1.914)	0.164	-	-
Tumor location	(left-sided vs. Right-sided)	0.642 (0.407–1.013)	0.057	0.578 (0.361–0.924)	0.022
Chemotherapy regimen	(Irinotecan vs. Oxali-based)	0.784 (0.541–1.135)	0.197	-	-
Number of cycles	-	0.911 (0.847–0.980)	0.012	0.915 (0.849–0.986)	0.020
Liver metastasis	(Yes vs. No)	0.817 (0.588–1.136)	0.230	-	-
Peritoneum metastasis	(Yes vs. No)	0.952 (0.691–1.313)	0.766	-	-
Resection of the primary tumor	(Yes vs. No)	0.426 (0.207–0.877)	0.014	0.476 (0.228–0.994)	0.048
Maintenance treatment	(Yes vs. No)	0.496 (0.353–0.698)	0.0001	0.536 (0.377–0.763)	0.001

## Discussion

This retrospective, observational study, which is the first of its kind from southern Morocco, was conducted to analyze the characteristics of our population and assess the efficacy and safety of treatment with bevacizumab in mCRC.

The introduction of bevacizumab into the treatment regimen has significantly modified the management of mCRC. Its use in conjunction with chemotherapeutic regimens has quickly become the standard of care in first- and second-line treatment [5-8]. Bevacizumab is a humanized monoclonal IgG1 antibody that inhibits tumor neovascularization and endothelial cell response associated with tumor permeability, proliferation, and migration [9]. The first trial that evaluated bevacizumab in mCRC, the AVF2107 trial, demonstrated its efficacy in combination with 5-FU and irinotecan with an improvement in OS of 4.7 months, leading to its approval by the Food and Drug Administration [10]. Bevacizumab was expected to have an activity as a single agent by reducing the blood vessel density within tumors. However, it provided a modest response rate (RR) contrary to its combination with chemotherapy, which demonstrated significant efficacy in terms of OS, PFS, and RR [11]. Survival rates (OS) have been raised from 8 to 10 months with a solitary drug, extending to more than 18 to 24 months when using combinations of chemotherapeutic agents, and peaking up to 34 months when bevacizumab has been introduced [12].

Age is a principal risk factor for CRC. This type of cancer is uncommon before the age of 40 years. Its incidence rises notably from age 40 to 50 years, with age-specific incidence rates increasing with each following decade. Data from the U.S. Surveillance, Epidemiology, and End Results database and other Western cancer registries indicate that CRC incidence is rising in the under-50-year age group and declining in older age groups [13]. The median age within our population was 55 years. A similar median age of 52 years was found in a study conducted in the eastern region of Morocco, specifically in Oujda [14].

CRC is heterogeneous in its tumor type, with pathogenesis determined by anatomical location, particularly the distinction between the right and left sides of the colon. Response to treatment varies considerably between these distinct tumor entities. Right-sided tumors often present with mutations in the DNA mismatch repair pathway and generally have a flat histology. Conversely, left-sided tumors often present with mutations in the chromosomal instability pathway, involving mutations in *KRAS*, *APC*, *PIK3CA*, and *p53*, and have a polypoid-like morphology [15]. In our study, the left side accounted for 83.2% of cases, while the right side represented only 16.6% of cases. These findings align with the results of other studies [12,14].

The majority of malignant tumors in the colon and rectum are carcinomas, of which 90% are adenocarcinomas. Signet ring cell carcinomas constitute an aggressive subtype of adenocarcinomas associated with an unfavorable overall prognosis. Mucinous carcinomas represent approximately 11-17% of all CRCs. This histological type has a predilection for the right side of the colon and may exhibit low sensitivity to initial chemotherapy [16]. In our study, adenocarcinoma constituted the most common histology (84.6%), followed by mucinous (14%) and signet ring cells (1.2%) histologies, which is similar to the data reported in the literature [16]. The department had limited information regarding the *RAS* mutation profile and other molecular characteristics due to the lack of other targeted therapies and the high cost of these analyses. Thus, we have to better investigate the molecular aspect in the future and request *RAS* and microsatellite instability (MSI) status for an effective therapeutic strategy.

Common sites for distant metastasis involve the liver and peritoneum. Roughly 20% of patients display synchronous metastases, with the liver being the primary location. Peritoneal metastases were identified in 25% of patients, which implied an inferior prognosis in comparison to metastases occurring in other locations [17]. We observed a liver metastasis rate of nearly 61%, which closely aligns with the 73% reported by Hugen et al. The elevated prevalence of adenocarcinoma within our cohort likely contributes to the heightened occurrence of liver metastases [18].

Regarding chemotherapy combinations used with bevacizumab, several studies have compared the FOLFOX with FOLFIRI protocols in combination with bevacizumab. Notably, Colucci et al. demonstrated that there is no disparity in RR (31% vs. 34%), OS (14 vs. 15 months), or PFS (7 months in both regimens) for individuals undergoing either regimen. Both combination therapies appeared to be effective as initial treatment for advanced CRCs [19]. The BEAT study reported the benefits of adding bevacizumab to various chemotherapy protocols. This study showed that bevacizumab improved RR, OS, and PFS when combined with 5-fluorouracil/leucovorin (LV5FU), FOLFIRI, FOLFOX, or XELOX. Tri-chemotherapy can also be utilized, as indicated by the TRIBE study for patients with *RAS* mutation, good performance status, and those who are fit for this regimen [20]. Capecitabine mono-chemotherapy, when combined with bevacizumab, is an interesting option for elderly and vulnerable individuals, as suggested by the AVEX study. This combination of bevacizumab and capecitabine has been shown to be an efficient and well-tolerated treatment regimen for elderly patients dealing with mCRC [21].

XELOX combined with bevacizumab was the most administered regimen in first-line chemotherapy (54.3%), aligning with findings from a real-world study conducted in the United Kingdom and Romania [22,23].

The PFS of nine months (interquartile range (IQR) = 7.58-10.15) noted in patients treated with first-line bevacizumab was nearly comparable to the findings of other observational studies (Romania: 8.4, India: 7.13, Oujda: 13) [12,14,23] and some clinical trials involving more interventions (9.3-17 months) [7-8].

The median OS of 14 months (IQR = 11.99-16.00) was comparatively shorter than other observational studies such as BRiTE (22.9 months) [24], BEAT (22.7 months) [25], and those reported in similar retrospective studies (Romania: 17.7, India: 18.5, Oujda: 22) [12,14,23]. Nevertheless, variations in study design and population demographics could also contribute to the disparities in OS rates.

In our study, the resection status of the primary tumor in mCRC patients had a significant impact on PFS and OS of patients treated with bevacizumab. A meta-analysis of seven studies involving 2,760 patients found that the resection group had a longer OS after bevacizumab treatment than the non-resection group (24.6 months vs. 17.5 months, respectively). Furthermore, for patients who underwent primary tumor resection, OS was significantly improved when bevacizumab was added to chemotherapy compared to chemotherapy alone [26]. Moreover, tumor location was a significant prognostic factor in OS, which aligns with the literature findings. A recent meta-analysis comprising 21 studies indicated that treatments based on bevacizumab are more effective in patients with left-sided mCRC in comparison to those with right-side tumors. Moreover, right-sided CRC is typically characterized by poorly differentiated, mucinous, signet ring histology with MSI and a mutation in *RAS*/*RAF*, in contrast to the left side [14].

The safety profile of bevacizumab met our expectations and showed good tolerance among our study population. Hypertension is the most common adverse event associated with bevacizumab, according to several studies. Its incidence varies from 19% to 34% for grades 1 and 2. Grade 4 was reported in fewer than 1% of cases and caused treatment interruption [27]. It is often explained by a mechanism of vascular resistance induced by bevacizumab. It was reported in 1.2% of our patients.

Our study limitations include the unavailability of the molecular profile, particularly for *RAS* mutation and MSI status, as well as the absence of other anti-EGFR treatments. Additionally, the retrospective nature of this study and the unresectable CRC status of the study population may have potentially negatively impacted our data, especially regarding survival.

## Conclusions

Chemotherapy combination with bevacizumab is considered the cornerstone of mCRC treatment in our region. This approach is favored due to the unavailability of treatments targeting EGFR, immunotherapy, or the characterization of RAS and MSI tumors. With the new healthcare and social security systems, easier access to expensive treatments and molecular pathology tests is currently possible. It is important to highlight that real-world data can offer valuable insights into the daily clinical practice of medical oncology. Thus, in this study, PFS showed more favorable outcomes compared to other studies, while OS was relatively shorter. However, the safety profile of bevacizumab was largely concordant with our expectations and those from the literature.
